# Fruit and vegetable intake and risk of prediabetes and type 2 diabetes: results from a 20-year long prospective cohort study in Swedish men and women

**DOI:** 10.1007/s00394-022-02871-6

**Published:** 2022-04-18

**Authors:** Afroditi Alexandra Barouti, Per Tynelius, Anton Lager, Anneli Björklund

**Affiliations:** 1 Department of Molecular Medicine and Surgery, Karolinska Institutet, Stockholm, Sweden; 2 Department of Global Public Health, Karolinska Institutet, Stockholm, Sweden; 3 Center for Diabetes, Academic Specialist Center, Region Stockholm, Stockholm, Sweden

**Keywords:** Prediabetes, Type 2 diabetes, Fruits, Vegetables, Prospective cohort

## Abstract

**Purpose:**

To investigate the association between fruit and vegetable intake (FVI) and the risk of developing prediabetes and type 2 diabetes (T2D) in a Swedish prospective cohort study.

**Methods:**

Subjects were 6961 men and women aged 35–56 years old at baseline, participating in the Stockholm Diabetes Prevention Program cohort. By design, the cohort was enriched by 50% with subjects that had family history of diabetes. Anthropometric measurements, oral glucose tolerance tests and questionnaires on lifestyle and dietary factors were carried out at baseline and two follow-up occasions. Cox proportional hazard models were used to estimate hazard ratios with 95% CIs.

**Results:**

During a mean follow-up time of 20 ± 4 years, 1024 subjects developed T2D and 870 prediabetes. After adjustments for confounders, the highest tertile of total FVI was associated with a lower risk of developing T2D in men (HR 0.76, 95% CI 0.60–0.96). There was also an inverse association between total fruit intake and prediabetes risk in men, with the HR for the highest tertile being 0.76 (95% CI 0.58–1.00). As for subtypes, higher intake of apples/pears was inversely associated with T2D risk in both sexes, whereas higher intakes of banana, cabbage and tomato were positively associated with T2D or prediabetes risk in either men or women.

**Conclusion:**

We found an inverse association between higher total FVI and T2D risk and between higher fruit intake and prediabetes risk, in men but not in women. Certain fruit and vegetable subtypes showed varying results and require further investigation.

**Supplementary Information:**

The online version contains supplementary material available at 10.1007/s00394-022-02871-6.

## Introduction

The number of people with diabetes continues to increase worldwide, and by 2030, diabetes is projected to affect close to 600 million people and be the seventh most common cause of death [1, 2]. Prediabetes, defined as impaired fasting glucose (IFG) and/or impaired glucose tolerance (IGT), is not only a high-risk state for progression to type 2 diabetes (T2D) with 5–10% developing T2D every year, but is also per se associated with higher risk of cardiovascular disease (CVD), kidney and nerve damage [3]. In 2019, at least 374 million people were estimated to have prediabetes [1]. Most of them are asymptomatic for years and consequently unaware of their condition and its associated health risks [4], which underlines the need for identifying early measures of prevention.

Fruit and vegetable intake (FVI) has been proposed for the prevention of various chronic diseases including T2D [5], with the suggested beneficial effects attributed to their low-energy and nutrient-dense content [6]. However, findings from prospective studies have been inconsistent [7–29], with the latest meta-analysis by Halvorsen et al. showing a weak inverse association between FVI and T2D [30]. The majority of these studies investigated baseline measurements of FVI and have mainly included women, or men and women combined in the analyses (Supplemental Table 1). As for the risk of developing prediabetes, there are no studies to our knowledge that have prospectively investigated the relation to FVI separately from other dietary factors.

Given the inconsistent evidence on FVI and T2D and the aforementioned research gap on prediabetes, the aim of the current study was to investigate the association between FVI and the risk of prediabetes and T2D in a cohort of Swedish men and women.

## Methods

### Study design

The present study is part of the Stockholm Diabetes Prevention Program (SDPP), a prospective cohort study that comprised men and women 35–56 years old at baseline, without previously diagnosed diabetes, from five municipalities in Stockholm County. The design of the study has been described elsewhere [31]. In brief, men and women of appropriate age, residing in the selected study areas, participated in a baseline survey during 1992–1994 for men and 1996–1998 for women (Supplementary Fig. 1). Subjects that already had diabetes, gave incomplete responses, were born outside of Sweden or had unclear or insufficient family history of diabetes (FHD), defined as at least one first or two second degree relatives with T2D, were excluded from further investigation. By study design, the sample was enriched by ~ 50% with subjects having either clear negative or positive FHD to examine the impact of diabetes heredity. Participants of the baseline survey were invited to a first follow-up after 8–10 years, in 2002–2004 for men and in 2004–2006 for women, and to a second follow-up in 2014–2017 (9–15 years after the first follow-up). A health examination, including anthropometric and blood pressure measurements and an oral glucose tolerance test (OGTT), as well as a questionnaire on lifestyle factors and a food frequency questionnaire (FFQ) were carried out at baseline and both follow-up occasions.

### Study population

The current study included all subjects who came to baseline (*n* = 7948), excluding those that were diagnosed with diabetes at the baseline health examination (*n* = 128), those diagnosed with type 1 diabetes during the total follow-up period (*n* = 16) and those with incomplete or missing data on FVI and potential confounders (*n* = 841) at baseline, resulting in a total sample of 6961 participants. For the analyses where prediabetes was the outcome, we further excluded those that were diagnosed with prediabetes at baseline (*n* = 386). Those with a prediabetes diagnosis preceding that of T2D contributed with person-time until the date of prediabetes diagnosis; the rest of T2D cases were excluded from prediabetes analyses (*n* = 578). The total sample size for prediabetes analyses was 5997. We did not perform separate analyses for the subjects that progressed from prediabetes to T2D, since their number was not large enough. All participants gave their informed consent and the study was approved by the Regional Ethics Review Board of Stockholm (2013/1982-31/2, 2018/2345-32).

### Assessment of diet and confounders

The FFQ of this study included initially 49 food items in the baseline survey of men in 1992–1994 and was later updated to include 54 items in the baseline survey of women in 1996–1998, in which, for example, a combined question for similar food items was divided into separate questions. This FFQ was originally created to assess fat intake as well as fiber intake from cereals and fruit and vegetables (FV), and therefore it did not include all available food groups and food items. It has been validated for fiber and fat intake by a 7-day weighed dietary record in a sample of 35–54 years old Swedish participants before the start of the SDPP study [32]; the validation showed good precision for total fiber intake but not for fat intake. According to the 1997–1998 national survey of the Swedish Food Agency that used a 7-day dietary record for dietary assessment [33], the most highly consumed fruits and vegetables during that period in Sweden were the ones included in this FFQ making up for 70–73% of the total daily FVI. The individual FV items included were apple/pear, banana, orange/satsumas/clementine, grapes, carrot, cabbage, tomato, peas/green beans and green salad (composed of lettuce, iceberg lettuce and Chinese cabbage), which were all assessed in both versions of the FFQ. Potatoes were excluded from vegetables due to their different nutrient content. The FFQ had eight frequency response options ranging from seldom/never to ≥ 4 times/day. Daily FVI in grams was calculated from the frequency responses combined with standard portion sizes/servings given by the Swedish National Food Agency and was then categorized into tertiles of intake, with the first tertile being characterized by lower consumption, the second by moderate and the third by higher consumption. It should be also noted that, as the FFQ was purposely limited to specific food items, it was not possible to calculate total energy intake. The FFQ was administered at baseline, follow-up 1 and follow-up 2 and it was completed while the subjects were waiting for the two-hour glucose ingestion of the OGTT, i.e., before any new diagnosis of prediabetes/T2D was made.

Confounders were selected based on established risk factors for diabetes and were updated in every follow-up. Family history of diabetes was categorized as either positive or negative. BMI was calculated by measurements of weight and height at every health examination (kg/m^2^) and subjects were categorized in three BMI groups according to WHO cut-offs [30]: normal- and underweight (BMI < 25), overweight (BMI 25–29.9) and obese (BMI > 30); underweight was only 0.7% of the total sample and was grouped together with normal weight. Leisure time physical activity during the last year was categorized into four groups as sedentary, moderately active, regular exercise, and regular exercise with extra training. Smoking was categorized into three groups: never, former and current smoker. Hypertension was categorized as yes or no according to blood pressure measurements and concurrent treatment (yes: systolic blood pressure ≥ 140 mm Hg or diastolic blood pressure ≥ 90 mm Hg and/or anti-hypertensive treatment; no: blood pressure ≤ 140/90 mm Hg and no treatment of hypertension). Education was categorized into three groups as low (elementary school and junior high school), middle (senior high school, technical and vocational school) and high (university and other training). Socioeconomic index (SEI) groups according to Statistics Sweden: unskilled/skilled manual workers— low-level non-manual workers; medium- and high-level non-manual workers; self-employed and farmers. Dietary covariates like wholegrain intake, that has been previously associated with lower T2D/prediabetes risk in this study population [34], and yoghurt/sour milk intake have also been included (see “Statistical analysis”).

### Classification of outcomes

A standard 75 g OGTT was carried out at baseline and both follow-up examinations, after an overnight fast in the morning. Concentrations of venous plasma glucose were analyzed in duplicate by a glucose oxidase method using a Yellow Springs Glucose Analyzer (Yellow Springs, OH, USA).

After the OGTT, subjects were categorized according to the 1999 World Health Organization criteria [35]. Specifically, normal glucose tolerance (NGT) was defined as fasting plasma glucose < 6.1 and 2 h glucose < 7.8 mmol/L; impaired fasting glucose was defined as fasting plasma glucose 6.1–6.9 and 2 h glucose < 7.8 mmol/L; impaired glucose tolerance was fasting plasma glucose < 6.1 and 2 h glucose 7.8–11.0 mmol/L; and type 2 diabetes was defined as fasting plasma glucose ≥ 7.0 and/or 2 h glucose ≥ 11.1 mmol/L. Those with a fasting plasma glucose of 6.1–6.9 mmol/L and 2 h glucose of 7.8–11.0 mmol/L were defined as having both IFG and IGT. Prediabetes was defined as having either IFG or IGT or both.

Data from the Stockholm Regional Health Care Data Warehouse (VAL; Vårdanalysdatabasen) and the Swedish National Diabetes Register (NDR) were used to ascertain T2D diagnosis of subjects lost to follow-up until the end of the 2nd follow-up (01 January 2018). The VAL-database covers almost all health care in Stockholm County including data from hospital inpatient care, specialist open care, primary care and data on collected prescribed medications [36, 37]. In NDR, which initiated in 1996 and has been described elsewhere [38], each patient provides informed consent for inclusion in the register and practically all patients with a confirmed diabetes diagnosis in Sweden are included. However, neither of the two registers provide information on prediabetes. Death was ascertained by the Swedish Population Register.

### Statistical analysis

Men and women were included and followed separately in SDPP and were therefore analyzed separately. Cox proportional hazards models with time-varying covariates were used to investigate the association between FVI and risk of prediabetes and T2D. Hazard ratios (HR) and 95% confidence intervals (CI) were calculated for each tertile of FVI with the lowest tertile as reference category. Separate analyses were performed for the two outcomes (prediabetes, T2D). The proportional hazard assumption was assessed with the help of Schoenfeld residuals. The cohort at risk included those free of the primary outcome at any time point. Each participant contributed with person-time of follow-up from baseline to the date of diagnosis (either prediabetes or T2D), death or the end of the 2nd follow-up (01 January 2018), whichever occurred first.

In the main analysis, we investigated associations between daily intake of total fruit, total vegetables and total fruit and vegetables combined and risk of prediabetes or T2D. Subtypes of fruit and vegetables were investigated in secondary analyses. Tertiles of cumulative averages of intake were used for all dietary covariates (FVI and other potential confounders) [39]; for example, for an individual with newly diagnosed T2D at the 2nd follow-up, the average FVI from baseline and 1st follow-up was calculated during the first risk period, whereas the average FVI from 1st and 2nd follow-up was used for the second risk period.

Analyses were adjusted as follows: Model 1 was adjusted for unmodifiable confounders: age and FHD; Model 2: adjusted as for Model 1 plus physical activity, smoking, education, SEI, hypertension, alcohol intake, total wholegrain intake and total yoghurt/sour milk intake, as well as total fruit intake for analyses on total vegetables and vice versa; Model 3 was adjusted as for Model 2 plus BMI. BMI can be considered both a confounder affecting FVI and prediabetes/T2D risk and a mediator being in the causal pathway of this relation; we, therefore, chose to present the hazard ratios with and without BMI. Further adjustments of Model 2 for other known confounders that were measured in the SDPP cohort, like snuffing, processed meat products, total dairy or coffee intake, did not change the estimates and were not included in the model. Non-dietary covariates were updated at each follow-up period. Tests for interaction with sex were also performed. In sensitivity analyses, we investigated associations using only baseline measurements of FVI and confounders, and also proportional cause-specific hazard models for prediabetes analyses, treating T2D as a competing event as proposed by Noordzij et al. [40]. The analyses were performed with STATA/IC Version 16.1.

## Results

**Fig. 1:**
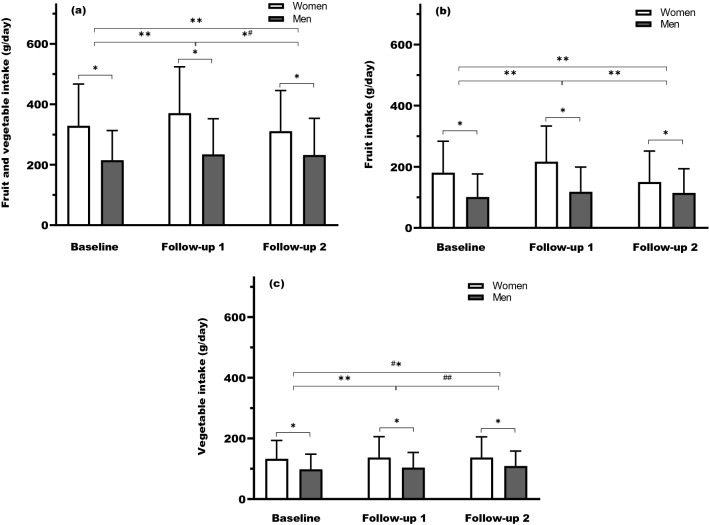
Total fruit and vegetable, total fruit and total vegetable intake of women and men at baseline (*n* = 6961), follow-up 1 (*n* = 5077) and follow-up 2 (*n* = 3626). Values are medians with vertical error bars representing 75% percentiles. *Significant differences between sexes using median test. Significant differences between follow-up occasions using Wilcoxon test in: both sexes **, only in women *^#^, only in men ^#^*, neither men nor women ^##^; significance level set at *P* < 0.05

The baseline characteristics of the study participants are presented in Table 1. Younger age, sedentary lifestyle, current smoking, lower education and manual work were more likely traits of those eating less fruit and vegetables at baseline, in both men and women. Women who consumed more fruit and vegetables were more likely to be overweight and obese at baseline than those with lower FVI, whereas men with lower FVI were more likely to have prediabetes at baseline. Family history of diabetes did not differ among tertiles of intake. FVI was significantly higher in women compared to men at baseline and both follow-up 1 and 2 (*P* < 0.001, Fig. 1). Median FVI of both men and women increased from baseline to follow-up 1 and then decreased for women at follow-up 2 whereas for men it remained stable (*P* < 0.001, Fig. 1).

**Table 1:** Characteristics of study participants by total fruit and vegetable intake at baseline (*N *= 6961)

	Total fruit and vegetable intake							
	Women				Men			
	Tertile 1	Tertile 2	Tertile 3	*P*^**a**^	Tertile 1	Tertile 2	Tertile 3	*P*^a^
Range (g/day)	< 254	254–415	> 415		< 160	160–278	> 278	
Median (g/day)	178	329	536		106	215	372	
n	1439	1436	1440		873	896	877	
Age (y)	48 (43–51)	49 (44–51)	49 (45–52)	< 0.001	46 (42–50)	47 (43–50)	47 (44–51)	0.006
Glucose tolerance at baseline								
Normal	1373 (95.4)	1378 (96.0)	1381 (95.9)	0.726	790 (90.5)	837 (93.4)	816 (93.0)	0.043
Prediabetes	66 (4.6)	58 (4.0)	59 (4.1)		83 (9.5)	59 (6.6)	61 (7.0)	
FHD	774 (53.8)	768 (53.5)	818 (56.8)	0.141	457 (52.3)	455 (50.8)	461 (52.6)	0.714
BMI (kg/m^2^)	24.2 (22.1–27.2)	24.6 (22.6–27.4)	25 (22.7–27.8)	< 0.001	25.8 (23.7–27.9)	25.5 (23.8–27.8)	25.6 (23.8–27.9)	0.172
BMI categories								
< 25	836 (58.1)	753 (52.4)	719 (49.9)	< 0.001	340 (38.9)	389 (43.4)	364 (41.5)	0.427
25–29.9	426 (29.6)	512 (35.7)	526 (36.5)		425 (48.7)	409 (45.7)	409 (46.6)	
> 30	177 (12.3)	171 (11.9)	195 (13.6)		108 (12.4)	98 (10.9)	104 (11.9)	
Physical activity								
Sedentary	228 (15.8)	145 (10.1)	105 (11.1)	< 0.001	127 (14.6)	75 (8.4)	57 (6.5)	< 0.001
Moderate	847 (58.9)	834 (58.1)	763 (53.0)		487 (55.8)	457 (51.0)	442 (50.4)	
Regular exersice	364 (25.3)	457 (31.8)	572 (39.7)		259 (29.7)	364 (40.6)	378 (43.1)	
Smoking								
Never	444 (30.9)	562 (39.1)	564 (39.2)	< 0.001	289 (33.1)	327 (36.5)	385 (43.9)	< 0.001
Former	492 (34.2)	520 (36.2)	580 (40.3)		286 (32.8)	351 (39.2)	336 (38.3)	
Current	503 (34.9)	354 (24.7)	296 (20.6)		298 (34.1)	218 (24.3)	156 (17.8)	
Education								
Low	484 (33.6)	411 (28.6)	384 (26.7)	< 0.001	317 (36.3)	255 (28.5)	258 (29.4)	< 0.001
Middle	493 (34.3)	467 (32.5)	472 (32.8)		397 (45.5)	399 (44.5)	388 (44.2)	
High	462 (32.1)	558 (38.9)	584 (40.6)		159 (18.2)	242 (27.0)	231 (26.3)	
SEI group								
1	406 (28.2)	365 (25.4)	383 (26.6)	< 0.001	317 (36.3)	254 (28.3)	262 (29.9)	< 0.001
2	407 (28.3)	370 (25.8)	339 (23.5)		155 (17.8)	128 (14.3)	142 (16.2)	
3	555 (38.6)	656 (45.7)	672 (46.7)		340 (38.9)	458 (51.1)	435 (49.6)	
4	71 (4.9)	45 (3.1)	46 (3.2)		61 (7.0)	56 (6.3)	38 (4.3)	
Hypertension	273 (19.0)	320 (22.8)	297 (20.6)	0.090	233 (26.7)	226 (25.2)	269 (30.7)	0.030
Wholegrain (g/day)	62 (32–92)	85 (54–131)	106 (69–168)	< 0.001	52 (26–87)	72 (40–119)	99 (62–163)	< 0.001
Yoghurt/sour milk (g/day)	53 (18–178)	90 (35–250)	178 (35–250)	< 0.001	35 (0–90)	70 (18–180)	90 (18–250)	< 0.001
Fruit (g/day)	79 (45–113)	186 (146–231)	336 (261–432)	< 0.001	41 (25–64)	109 (70–144)	217 (159–293)	< 0.001
Vegetables (g/day)	82 (52–114)	137 (105–174)	211 (150–298)	< 0.001	53 (31–79)	104 (77–137)	157 (112–218)	< 0.001

Values are numbers (percentages) or medians (interquartile range). SEI groups: 1, manual workers; 2, low-level non-manual workers; 3, medium- and high-level non-manual workers; 4, self-employed and farmers

*FHD* family history of diabetes, *SEI group* socioeconomic index group

^a^*P* values were calculated by median test for equality of medians for continuous variables and the Pearson chi-square test for categorical variables

During a mean follow-up time of 20 ± 4 years, a total of 1024 incident cases of T2D were documented (493 women and 531 men). Prediabetes was diagnosed in 386 subjects at baseline (183 women and 203 men) and 870 subjects during the total follow-up time (420 women and 450 men). The results of the Cox regression analyses using time-varying covariates and cumulative averages of dietary intake for categorizations to tertiles are presented in Table 2. Total FVI was inversely associated with the risk of developing T2D in men, and after adjusting for all confounders, the highest FVI tertile was associated with a 24% lower T2D risk compared to the lowest tertile of FVI (HR 0.76; 95% CI 0.60–0.96). Total FVI was also inversely associated with T2D risk in women in the age and FHD adjusted model, though not significantly after adjusting for additional confounders. Furthermore, total fruit intake was associated with a lower risk of developing prediabetes in men in the fully adjusted multivariate model (HR of the third tertile 0.76; 95% CI 0.58–1.00). In women, higher total FVI was positively associated with the risk of developing prediabetes in the second model (HR 1.33; 95% CI 1.01–1.74), but the association was no longer significant after further adjusting for BMI (HR 1.25; 95% CI 0.95–1.64). No significant associations were seen between total vegetable intake and the risk of developing T2D or prediabetes.

**Table 2:** Hazard ratios (95% CI) estimated by cox regression analysis with time varying covariates for the association between tertiles of fruit and vegetable intake and risk of T2D/prediabetes at follow-up (*N *= 6961 and 5997 in T2D and prediabetes analyses respectively)

	Women			Men			*P*-interaction^**a**^
	Tertile 1	Tertile 2	Tertile 3	Tertile 1	Tertile 2	Tertile 3	
**Type 2 diabetes (from NGT/prediabetes)**							
Total FVI (median)	211	360	560	125	234	397	
Cases	187	158	145	212	168	138	
Person-years	29269	26982	26710	19073	18669	19096	
Model 1	1.00	0.88 (0.71, 1.08)	0.81 (0.65, 1.00)	1.00	0.81 (0.66, 0.99)	0.62 (0.50, 0.77)	0.166
Model 2	1.00	1.03 (0.83, 1.30)	1.00 (0.79, 1.27)	1.00	0.90 (0.73, 1.12)	0.77 (0.61, 0.97)	0.156
Model 3	1.00	0.98 (0.78, 1.22)	0.93 (0.74, 1.19)	1.00	0.92 (0.74, 1.14)	0.76 (0.60, 0.96)	0.243
Total fruit (median)	100	204	354	49	116	237	
Cases	185	162	144	204	169	151	
Person-years	29546	27130	26716	19465	18950	19070	
Model 1	1.00	0.89 (0.72, 1.10)	0.79 (0.64, 0.99)	1.00	0.82 (0.67, 1.01)	0.75 (0.56, 0.86)	0.542
Model 2	1.00	0.99 (0.79, 1.25)	0.85 (0.67, 1.10)	1.00	0.96 (0.77, 1.19)	0.90 (0.71, 1.14)	0.669
Model 3	1.00	0.96 (0.76, 1.20)	0.80 (0.63, 1.03)	1.00	0.93 (0.75, 1.16)	0.84 (0.66, 1.07)	0.751
Total vegetables (median)	82	140	238	53	106	181	
Cases	186	139	166	208	163	147	
Person-years	28855	27542	27002	18978	19112	18975	
Model 1	1.00	0.78 (0.63, 0.98)	0.94 (0.77, 1.17)	1.00	0.77 (0.62, 0.94)	0.71 (0.57, 0.87)	0.091
Model 2	1.00	0.90 (0.71, 1.13)	1.26 (0.99, 1.60)	1.00	0.94 (0.76, 1.17)	0.91 (0.72, 1.15)	0.031
Model 3	1.00	0.87 (0.69, 1.10)	1.24 (0.98, 1.56)	1.00	1.03 (0.83, 1.29)	0.96 (0.76, 1.21)	0.023
**Prediabetes (from NGT)**							
Total FVI (median)	202	353	551	126	236	397	
Cases	125	136	141	162	139	126	
Person-years	26692	24435	24140	15953	15761	15997	
Model 1	1.00	1.14 (0.89, 1.45)	1.19 (0.94, 1.52)	1.00	0.85 (0.67, 1.06)	0.74 (0.59, 0.93)	0.011
Model 2	1.00	1.24 (0.95, 1.61)	1.33 (1.01, 1.74)	1.00	0.89 (0.70, 1.13)	0.82 (0.64, 1.06)	0.004
Model 3	1.00	1.16 (0.89, 1.51)	1.25 (0.95, 1.64)	1.00	0.88 (0.69, 1.12)	0.81 (0.62, 1.04)	0.007
Total fruit (median)	95	199	345	48	116	238	
Cases	126	154	128	157	156	125	
Person-years	26801	24633	24236	16218	15892	16128	
Model 1	1.00	1.25 (0.98, 1.59)	1.04 (0.81, 1.33)	1.00	0.97 (0.78, 1.22)	0.75 (0.59, 0.95)	0.094
Model 2	1.00	1.32 (1.02, 1.71)	1.06 (0.80, 1.42)	1.00	1.07 (0.84, 1.37)	0.80 (0.61, 1.05)	0.035
Model 3	1.00	1.26 (0.97, 1.64)	1.01 (0.76, 1.35)	1.00	1.04 (0.82, 1.33)	0.76 (0.58, 1.00)	0.038
Total vegetables (median)	79	138	236	54	107	181	
Cases	132	140	134	162	125	142	
Person-years	26275	24945	24434	15964	16028	15925	
Model 1	1.00	1.13 (0.87, 1.43)	1.09 (0.86, 1.38)	1.00	0.77 (0.61, 0.97)	0.88 (0.70, 1.10)	0.080
Model 2	1.00	1.13 (0.87, 1.46)	1.20 (0.92, 1.58)	1.00	0.79 (0.62, 1.02)	1.03 (0.80, 1.34)	0.047
Model 3	1.00	1.12 (0.87, 1.45)	1.16 (0.89, 1.52)	1.00	0.85 (0.66, 1.09)	1.08 (0.84, 1.40)	0.128

*T2D* type 2 diabetes, *NGT* normal glucose tolerance, *FVI* fruit and vegetable intake

^a^Test for interaction with sex. Median intakes in g/day. Model 1 adjusted for age and family history of diabetes; Model 2 adjusted as for Model 1 plus education, socioeconomic index group, high blood pressure, physical activity, smoking, alcohol, wholegrain intake, yoghurt/sour milk intake, total fruit intake for total vegetable intake analyses and vice versa; Model 3 adjusted as for Model 2 plus BMI

We repeated the main analyses using only the baseline measurements of total fruit, total vegetable and total fruit and vegetable intake and confounders (Supplementary Table 2). The inverse association between higher FVI and risk of T2D in men was also shown in these analyses (HR 0.77; 95% CI 0.62–0.97). Higher fruit intake in men was inversely associated with prediabetes risk (HR 0.81 for the highest tertile in Model 3), though confidence intervals were relatively broad (95% CI 0.63–1.03). We found no associations in women, in line with the time-varying analyses. We also repeated the analyses on prediabetes using proportional cause-specific hazards models, where T2D cases were included as censored observations (data not shown). The previously shown association between fruit intake and prediabetes risk in men was similar but slightly attenuated (HR 0.78; 95% CI 0.60–1.02).

In secondary analyses, we investigated the associations between different subtypes of fruit and vegetables and the risk of developing T2D or prediabetes, after adjusting for all confounders (Table 3). Higher intake of apples/pears was associated with lower risk of T2D in both men and women (HR 0.70, 95% CI 0.55–0.90 in men; HR 0.64, 95% CI 0.47–0.88 in women). In contrast, the highest tertile of tomato intake had a positive association with T2D risk in women (HR 1.37; 95% CI 1.03–1.82). As for the risk of developing prediabetes, we observed positive associations with higher intake of banana in men (HR 1.35; 95% CI 1.03–1.78) and cabbage in women (HR 1.45; 95% CI 1.12, 1.87).

**Table 3:** Hazard ratios (95% CI) estimated by cox regression analysis with time varying covariates for the association between tertiles of intake of fruit and vegetable subtypes and risk of T2D/prediabetes at follow-up in fully adjusted models (*N* = 6961 and 5997 in T2D and prediabetes analyses respectively)

	Women			Men			*P*-interaction^**a**^
	Tertile 1	Tertile 2	Tertile 3	Tertile 1	Tertile 2	Tertile 3	
**Type 2 diabetes (from NGT/prediabetes)**							
Apples/pears (median)	27	107	219	9	45	125	
Cases	204	222	66	245	145	134	
Person-years	30599	40081	13125	20397	20383	16869	
Model 3	1.00	0.82 (0.66, 1.01)	0.64 (0.47, 0.88)	1.00	0.67 (0.53, 0.84)	0.70 (0.55, 0.90)	0.291
Oranges/satsumas/clementines (median)	13	49	125	9	27	71	
Cases	176	137	179	224	168	132	
Person-years	29545	29316	24994	23072	17700	16875	
Model 3	1.00	0.97 (0.76, 1.23)	1.22 (0.96, 1.54)	1.00	1.09 (0.87, 1.35)	0.96 (0.75, 1.23)	0.155
Grapes (median)	0	9	18	0	9	18	
Cases	176	218	97	208	195	121	
Person-years	31819	33012	18851	22633	21090	13910	
Model 3	1.00	1.18 (0.96, 1.45)	0.98 (0.75, 1.27)	1.00	0.94 (0.76, 1.15)	1.06 (0.83, 1.35)	0.27
Banana (median)	7	38	90	7	26	71	
Cases	230	134	128	238	168	118	
Person-years	31568	27946	24367	23517	18800	15397	
Model 3	1.00	0.86 (0.69, 1.08)	0.85 (0.67, 1.07)	1.00	0.93 (0.76, 1.15)	1.10 (0.85, 1.41)	0.809
Carrot (median)	6	31	73	6	18	58	
Cases	206	135	150	222	196	104	
Person-years	31609	29627	22513	22064	22112	13430	
Model 3	1.00	0.91 (0.71, 1.15)	1.28 (0.99, 1.65)	1.00	0.98 (0.79, 1.20)	1.19 (0.90, 1.58)	0.178
Green salad (median)	11	31	44	6	24	44	
Cases	218	132	142	217	199	108	
Person-years	31192	30574	22133	19061	22569	16056	
Model 3	1.00	0.79 (0.62, 1.00)	0.89 (0.68, 1.17)	1.00	1.01 (0.81, 1.26)	0.79 (0.58, 1.08)	0.014
Cabbage (median)	5	10	30	2	10	27	
Cases	266	77	149	298	87	137	
Person-years	43161	16322	24173	29809	10608	16985	
Model 3	1.00	0.85 (0.65, 1.11)	1.03 (0.82, 1.30)	1.00	1.01 (0.78, 1.30)	1.04 (0.82, 1.31)	0.447
Tomato (median)	25	60	92	18	40	70	
Cases	187	188	117	279	108	136	
Person-years	32063	31845	20019	23881	15357	18288	
Model 3	1.00	1.16 (0.92, 1.45)	1.37 (1.03, 1.82)	1.00	0.70 (0.54, 0.90)	0.82 (0.63, 1.07)	<0.001
Peas/green beans (median)	7	14	43	7	14	36	
Cases	232	104	156	267	118	138	
Person-years	35175	25180	23513	26309	15089	16275	
Model 3	1.00	0.74 (0.58, 0.95)	1.00 (0.80, 1.27)	1.00	0.93 (0.74, 1.17)	1.03 (0.81, 1.30)	0.104
**Prediabetes (from NGT)**							
Apple/pear (median)	27	107	219	9	45	125	
Cases	135	204	72	175	155	110	
Person-years	28214	35783	12061	17452	16473	14456	
Model 3	1.00	1.08 (0.84, 1.39)	1.15 (0.82, 1.60)	1.00	0.99 (0.78, 1.27)	0.80 (0.61, 1.07)	0.013
Orange/satsumas/ clementine (median)	13	49	125	9	27	71	
Cases	104	194	115	195	118	127	
Person-years	26935	26886	22292	19108	14735	14552	
Model 3	1.00	1.70 (1.30, 2.22)	1.16 (0.86, 1.43)	1.00	0.72 (0.56, 0.93)	0.82 (0.63, 1.07)	<0.001
Grapes (median)	0	9	18	0	9	18	
Cases	164	139	108	185	137	117	
Person-years	29080	29771	17092	18995	17701	11672	
Model 3	1.00	0.78 (0.61, 0.99)	1.10 (0.85, 1.43)	1.00	0.72 (0.57, 0.92)	1.00 (0.78, 1.31)	0.488
Banana (median)	7	38	90	7	26	71	
Cases	127	180	107	165	155	119	
Person-years	28315	25680	22121	19173	15727	13550	
Model 3	1.00	1.45 (1.14, 1.87)	1.07 (0.80, 1.43)	1.00	1.21 (0.95, 1.54)	1.35 (1.03, 1.78)	0.011
Carrot (median)	6	31	73	6	21	58	
Cases	118	193	100	188	137	110	
Person-years	28345	27151	20523	19484	17264	11623	
Model 3	1.00	1.50 (1.15, 1.95)	1.02 (0.74, 1.40)	1.00	0.86 (0.68, 1.09)	1.23 (0.92, 1.63)	<0.001
Green salad (median)	11	30	44	9	24	44	
Cases	121	201	93	209	125	104	
Person-years	27911	28287	19956	22241	12172	14009	
Model 3	1.00	1.97 (1.51, 2.56)	1.05 (0.74, 1.49)	1.00	1.07 (0.81, 1.42)	0.80 (0.58, 1.10)	0.006
Cabbage (median)	2	10	30	2	7	27	
Cases	178	79	154	220	70	147	
Person-years	39044	14975	21977	24682	8958	14547	
Model 3	1.00	1.22 (0.91, 1.62)	1.45 (1.12, 1.87)	1.00	0.97 (0.73, 1.30)	1.19 (0.93, 1.52)	0.086
Tomato (median)	25	60	100	18	37	70	
Cases	178	142	94	163	141	135	
Person-years	29050	29018	18114	19256	13217	15811	
Model 3	1.00	0.70 (0.55, 0.90)	1.08 (0.79, 1.48)	1.00	1.26 (0.95, 1.66)	1.12 (0.83, 1.51)	0.012
Peas/green beans (median)	7	14	43	7	14	36	
Cases	154	134	124	195	110	134	
Person-years	31086	23060	21961	20702	13251	14447	
Model 3	1.00	0.97 (0.75, 1.25)	1.00 (0.76, 1.31)	1.00	1.02 (0.79, 1.32)	1.09 (0.84, 1.40)	0.617

*T2D* type 2 diabetes, *NGT* normal glucose tolerance

^a^Test for interaction with sex. Median intakes in g/day. Model 3 adjusted for age, family history of diabetes, education, socioeconomic index group, high blood pressure, physical activity, smoking, alcohol, wholegrain intake, yoghurt/sour milk intake, total fruit intake for vegetable subtype analyses and vice versa, and BMI

## Discussion

Our study’s distinctive feature is that it allowed to prospectively investigate not only the risk of developing T2D but also the risk of developing prediabetes from normal glucose tolerance. We found that higher fruit intake was associated with lower risk of prediabetes in men. To our knowledge, there are no other prospective studies that investigated FVI separately from other dietary factors in relation to prediabetes risk. The Rotterdam Study investigated prospectively a plant-based eating pattern in 6798 participants and found that higher adherence was associated with lower insulin resistance and prediabetes risk after 5.7 years of follow-up, though the association for prediabetes was no longer significant after adjusting for BMI [41]. Cross-sectional analyses of FVI in Chinese populations, either separately [42] or as part of dietary patterns [43, 44] reported inverse associations with the risk of prediabetes. Finally, Safabakhsh et al. investigated FVI in 150 prediabetes cases and 150 controls with NGT, and found an inverse association between total FVI and total fruit intake with prediabetes risk [45]. Considering the increasing evidence of kidney and nerve damages already at the pre-diabetic stage [3], possible measures of prevention, such as our findings on high fruit intake, should be further investigated in future prospective studies.

In addition, this study found that higher total FVI was associated with 24% lower risk of developing T2D from NGT or prediabetes in men. The latest meta-analysis of prospective cohort studies by Halvorsen et al. also found an inverse association between high intake of FV combined and T2D risk, though the effect was smaller (RR 0.93, 95% CI 0.89–0.98). Previous prospective studies have shown inconsistent results, presenting either inverse or no associations (Supplementary Table 1). Possible reasons for these inconsistencies could be related to the method of dietary assessment that was used. Cooper et al. reported in their meta-analysis that associations between FVI and T2D risk had a tendency to be weaker when intake was assessed with FFQ compared to other assessment methods such as 24 h recall [23]. FFQ can be prone to measurement error and recall bias, which can lead to underestimation of diet–disease associations [46]. In contrast, studies using objective biomarkers of FVI, such as vitamin C and carotenoids, have shown stronger inverse associations with T2D risk [47]. Furthermore, most prospective studies using repeated measurements of fruit or vegetable intake have shown significant inverse associations [7–10], whereas in studies with only one baseline measurement, the associations with T2D risk were often non-significant [13, 14, 17, 21–23, 25, 26, 28, 29]. Repeated dietary measurements and the use of cumulative averages have been used to better represent long-term intakes, take into account within-person variation and possibly decrease measurement error [39]. In our study, the inverse association between total FVI and T2D risk in men was significant and similar in both analyses, while other studies that compared the two methods (baseline intake versus repeated measurements of other dietary factors) have either shown similar or stronger diet-disease associations with repeated measurements [48, 49].

The inverse associations between higher total FVI and total fruit intake with T2D or prediabetes risk respectively, were found only in men. A statistically significant sex interaction was found for fruit intake (*P* = 0.038) but not for total FVI (*P* = 0.243). However, we cannot rule out that this was due to a lack of power in the analyses, or chance, as indicated by the relatively broad confidence intervals. Another explanation could be the difference in FVI between sexes, as women in our study had higher intake of both fruit and vegetables. Previous meta-analyses have demonstrated a non-linear dose–response association between fruit and/or vegetable intake and T2D risk [30, 50, 51]. Specifically, the relative risk of T2D decreased with the consumption of up to 200 g/day of fruit, and then increased with intakes above this level [30]; this might be attributed to the consequent higher intake of fructose from fruit, which has been linked to decreased insulin sensitivity [51]. In our study, women in the third tertile of fruit consumption had a median intake of 345 g/day (compared to men’s 238 g/day), and even though we did not assess or rely on absolute intakes in the current methodology, this could be one of the possible reasons for the difference in findings between men and women. Women in the third tertile of FVI had also higher BMI at baseline compared to women in the first tertile, while for men there were no differences in BMI among tertiles. Having a higher BMI could be a result of high energy intake, which could not be adjusted for in the models and may have affected the associations for women. Other studies have also reported different findings in men and women but these have been inconsistent, with either men or women showing inverse associations [8, 11, 18, 27].

Our secondary analysis on subtypes showed a possibly inverse association between intake of apples/pears and T2D risk in both men and women, which is also supported by findings of a recent meta-analysis of subtypes (RR per 100 g/day 0.90; 95% CI 0.83–0.97) [30]. Apples, which are consumed much more frequently in Sweden than pears [33], contain certain phytochemicals that are suggested to have beneficial effects on glucose metabolism, such as anthocyanins, quercetin and chlorogenic acid [8, 30]. In animal models, anthocyanins were found to enhance uptake and utilization of glucose in adipose tissue and muscle and reduce glucose production in the liver [52], and quercetin treatment was found protective against oxidative stress in pancreatic beta cells [53]. Chlorogenic acid, which is also found in coffee, has been suggested to have antidiabetic effect possibly by delaying intestinal glucose absorption and reducing hepatic glucose output [54]. In contrast to apples and pears, higher intakes of banana, cabbage and tomato may be associated with a higher risk of T2D or prediabetes in either men or women in our study. The effect of banana intake could be explained by the fruit’s high glycemic index, which has been associated with higher T2D risk in some studies [55], though previous prospective studies have shown varying associations between banana intake and T2D risk [8, 18, 56]. Ma et al. investigated cabbage intake and showed similar results to our findings, as they also found a positive association in women (Nurses’ Health Study I and II) but not in men (Health Professionals Follow-Up Study) with T2D risk [57]. The authors speculated that the positive association may be due to a pro-oxidant activity of dietary glucosinolates, a group of plant metabolites abundant in cruciferous vegetables, though these mechanisms are still complicated and conflicting [57]. It should be noted that a protective effect of fruit and vegetable intake on the development of prediabetes and T2D may be mediated by several other compounds and micronutrients they contain, like fiber [58], magnesium [59] and different antioxidants [60], and possibly in a synergistic manner, as greater FV variety has been associated with lower T2D risk [61].

Our study had several strengths; it was prospective, had a long duration of 20 years and used direct measurements for prediabetes assessment (OGTT). For T2D diagnosis, we used either direct measurements at each follow-up occasion or health care information from two different patient registers, which allowed to ascertain diabetes diagnosis even for participants that were lost to follow-up. We also used the majority of the participants that were free of the outcomes at baseline in our main analyses (89% of the available baseline study population had complete information on exposure and covariates), decreasing the risk of selection bias. Furthermore, the study included repeated measurements of the exposure and covariates when possible, taking into consideration within-person variation and potential changes over time. Finally, we have analyzed men and women separately, which allows for some extra insights in this research topic, since most of the available studies have used either only male or only female participants, or both combined in the analyses.

The main limitation of our study is that our FFQ was originally created and validated to assess fiber and fat intake only [32], and did not include all available individual fruit and vegetables, thus introducing measurement error. According to the 1997–1998 national survey of the Swedish Food Agency [33], which used 7-day weighed dietary records, the mean daily FVI in Stockholm region was actually lower than our study’s values, especially for women: 260 g/day for women and 180 g/day for men in the survey, compared to 330 g/day for women and 215 g/day for men in our baseline population. Compared to dietary records, assessment with FFQ is known to entail the risk of overestimating healthy foods and underestimating unhealthy foods (social desirability bias) [62], and considering that stronger healthy eating beliefs and higher weight control motivation are more common attributes of women than men [63, 64], overestimation of FVI by our female participants is, to some extent, not surprising. Nevertheless, since diet was assessed prospectively, reporting errors would likely be non-differential. In addition, FFQs are generally designed to assess the ranking of intakes within a study population rather than absolute intakes of foods and nutrients [62], and in our study, we used tertiles to decrease the risk of misclassification, which is higher the more and narrower the categories are. Another limitation of the study is that we could not adjust for total energy intake and additional foods related to T2D risk, like red meat and sugar-sweetened beverages (these were not included in the FFQ). It is likely that energy intake was partially accounted for indirectly, by adjusting for BMI and physical activity [65], but the occurrence of other unmeasured confounding cannot be excluded.

Finally, prediabetes cases could not be ascertained by the registers and therefore loss to follow-up may have affected these associations and played a role in the observed differences between prediabetes and T2D findings. We investigated, therefore, how follow-up rates differed among tertiles of intake (Supplementary Table 3), and found that the rates of loss to follow-up were significantly higher in women with low FVI at baseline compared to high FVI. As discussed in a previous study [31], women who did not participate in the first SDPP follow-up had a greater prevalence of obesity and lower prevalence of regular physical activity at baseline compared to those who participated. However, these differences were not found between male participants/non-participants and neither did attrition rates differ among FVI tertiles in men. Therefore, loss to follow-up is less likely to have affected the observed associations with prediabetes risk in men. Another possibility is that some results might be chance associations, as no adjustments were made for multiple comparisons. However, it is also possible that adjustments could have led to rejection of true associations and we have therefore cautiously interpreted our results with respect to the strength of the associations, consistency across models and in view of their supportiveness to previous observations.

In conclusion, the current study found an inverse association between higher total FVI and T2D risk and between higher fruit intake and prediabetes risk, in men but not in women. Our results also suggest that intake of certain subtypes, like apples and pears, may have a more favorable effect, while others may be associated with higher risk of T2D or prediabetes. Future prospective studies should further investigate the role of FV on the risk of developing prediabetes, the impact of different FV subtypes, as well as potential effect differences between sexes.

## Supplementary Information

Below is the link to the electronic supplementary material.

Supplementary file1 (DOCX 126 KB)

## Data Availability

Data described in the manuscript and analytic code will be made available upon request pending approval by the authors.
