# Supplementary material for: Fruit and vegetable intake and risk of prediabetes and type 2 diabetes: results from a 20-year long prospective cohort study in Swedish men and women
Source: Eur J Nutr. 2022 Apr 18;61(6):3175–87. doi: 10.1007/s00394-022-02871-6 (PMC9363331; doi:10.1007/s00394-022-02871-6)
Supplement: Supplementary file 1 — Supplementary file1 (DOCX 126 KB) [file 394_2022_2871_MOESM1_ESM.docx]

# **Supplementary Information**

| **Supplementary Table 1. Characteristics and reported associations from previous prospective cohort studies on fruit and vegetable intake and type 2 diabetes risk, categorized by type of exposure measurement.** | | | | | | | |
| --- | --- | --- | --- | --- | --- | --- | --- |
| **Study** | **Sample size and age range (years)** | **Follow-up (years)** | **Number of diabetes cases and outcome measure** | **Dietary assessment (assessment tool, measurement unit)** | **Confounders measured** | **Observed associations by exposure ^a^** | |
| **Studies with repeated measurements of FVI** | | | | | | | |
| Rayner et al. 2020 [7]  Australian Longitudinal Study on Women's Health  Australia | 9689 women  Age 50-55 | 15 | 959  Self-reported/ validation against hospital discharge data in a subset of the cohort | 80-item FFQ  Average intake in servings/day for total vegetables, total fruit and fruit juice | Age, country of birth, energy intake, highest educational qualification, employment status, years of follow-up, history of gestational diabetes, physical activity, cereal, high fiber bread, pasta and rice, discretionary foods, white bread, dairy, red and processed meat, fish, BMI, added sugar | RR (95% CI) lowest/highest quartile: | |
|  |  |  |  |  |  | Total fruit  Total vegetables | 1.22 (1.01, 1.48)  1.03 (0.84, 1.26) |
| Muraki et al. 2013 [8]  3 studies included: Nurses’ Health Study (NHS), Nurses’ Health Study II (NHS II), Health Professionals Follow-up Study (HPFS)  USA | NHS: 66,105 women  Mean age 49.9  NHS II: 85,104 women  Mean age 36.7  HPFS: 36,173 men  Mean age 52.3 | Up to 24  Person years  NHS: 1394127  NHS II: 1416111  HPFS: 654403 | NHS: 6358  NHS II: 3153  HPFS: 2687  Self-reported Confirmed by the National Diabetes Data Group criteria (before 1997), by ADA (after 1997) | 118 item FFQ  Cumulative average intake in servings/week for total fruit and individual fruits | Age, BMI, smoking, physical activity, family history of diabetes, menopausal status, post-menopausal hormone use, oral contraceptive use, ethnicity, total energy intake, multivitamin use, fruit juice consumption, alternate healthy eating index score | HR (95% CI) highest/lowest consumption level: | |
|  |  |  |  |  |  | Total fruit (NHS)  Total fruit (NHS II)  Total fruit (HPFS) | 0.90 (0.81, 0.99)  0.92 (0.78, 1.08)  0.90 (0.78, 1.04)  Pooled results: 0.90 (0.84, 0.97)  *corrected HRs after Erratum |
| Villegas et al. 2008 [9]  Shanghai Women’s Health Study  China | 64,191 women  Age 40-70 | 4.6 | 1608  Self-reported  Confirmed by ADA criteria | 77 item FFQ  Baseline or mean intake in g/day for total fruit, total vegetables and separate vegetable categories | Age, BMI, WHR, physical activity, smoking, alcohol, hypertension, total energy intake, meat intake, income level, education, occupational status | HR (95% CI) highest/lowest quintile: | |
|  |  |  |  |  |  | Total fruit  Total vegetables | 1.05 (0.90, 1.23)  0.72 (0.61, 0.85) |
| Bazzano et al. 2008 [10]  Nurses’ Health Study (1984 onwards)  USA | 71,346 women  Age 30-55 | 18 | 4529  Self-reported  Confirmed by National Diabetes Data Group criteria (before 1997) and by ADA criteria (after 1997) | 61 item FFQ  Cumulative average intake in servings/day for total fruit, total vegetables, FV combined, GLV, legumes, fruit juices | Age, BMI, physical activity, smoking, alcohol, hormone therapy, whole grain, nuts, processed meat, coffee, potatoes, soft drinks | HR (95% CI) highest/lowest quintile: | |
|  |  |  |  |  |  | FV combined  Total fruit  Total vegetables | 1.01 (0.90, 1.12)  0.90 (0.80, 1.00)  1.05 (0.94, 1.16) |
| **Studies with baseline measurements of FVI** | | | | | | | |
| Ahmed et al. 2020 [11]  Stockholm Public Health Cohort  Sweden | 14 718 men and 20 589 women  Age 25-84 | 4 | 319  Self-reported | Questionnaire (1 question for total fruit and 1 question for total vegetable intake: servings per day or week or month during the past 12 months)  Baseline intake in servings/day for fruit, vegetable, FV combined | Age, education, country of birth, BMI, smoking, alcohol, and physical activity | OR (95%CI) low/high intake (<2 vs ≥2 servings/d for F and V, <4 vs ≥4 servings/d for FV): | |
|  |  |  |  |  |  | FV combined ♂  FV combined ♀  Total fruit ♂  Total fruit ♀  Total vegetables ♂  Total vegetables ♀ | 1.17 (0.66, 2.08)  1.07 (0.73, 1.58)  1.04 (0.69, 1.59)  0.90 (0.65, 1.26)  1.62 (1.00, 2.61)  1.18 (0.82, 1.71) |
| Dow et al. 2019 [12]  The Australian Diabetes, Obesity and Lifestyle Study (AusDiab)  Australia | 6242 men and women  Age ≥ 25 years | 11.7 | 376  Standard oral glucose tolerance test | 80-item semi-quantitative FFQ  Baseline intake in servings/day for total fruit and total vegetables | Age, sex, education, smoking status, recreational physical activity, high triglycerides, low HDL cholesterol, family history of diabetes, energy intake, hypertension and waist circumference | HR (95%CI) strong vs weak adherence to recommendations: | |
|  |  |  |  |  |  | Total vegetable  Total fruit  for ♂ & ♀ combined | 0.83 (0.37,1.88)  0.68 (0.51, 0.91) |
| Khalili-Moghadam et al. 2018 [13]  Tehran Lipid and Glucose Study (TLGS)  Iran | 2139 men and women  Age 20-70 | 5.8 | 143  Fasting plasma glucose levels | 168 semi-quantitative FFQ  Baseline intake in serving/day for total fruit and total vegetables | Diabetes risk score: family history of diabetes, FPG concentrations, SBP, WHtR and TG/ HDL-C | HR (95% CI) highest/lowest tertile: | |
|  |  |  |  |  |  | Total vegetables  Total fruit  for ♂ & ♀ combined | 0.89 (0.57, 1.39)  0.75 (0.46, 1.22) |
| Chen et al. 2018 [14]  Singapore Chinese Health Study (SCHS)  Singapore | 45 411  participants,  Age 45-74 | 10.9 | 5207  Self-reported, validated by linkage with a nationwide hospital-based discharge database and supplementary questionnaire | 165-item FFQ  Baseline intake in g/2000 kcal per day for total vegetable and vegetable subtypes | Age, sex, dialect group, year of baseline interview, energy intake, physical activity, education, smoking, alcohol, soft drink, coffee, energy-adjusted intakes of red meat, poultry, fish, nuts and seeds, soya products and wholegrains, BMI, history of hypertension | HR (95% CI) highest/lowest quintile: | |
|  |  |  |  |  |  | Total vegetables  for ♂ & ♀ combined | 1.08 (0.98, 1.18) |
| Lv et al. 2017 [15]  The China Kadoorie Biobank Study (CKB)  China | 461 211 men and women  Age 30-79 | 7.2 | 8784  Linkage with local disease and death registries | Short qualitative FFQ  Baseline intake of FV combined | Age, sex, education, marital status, family history of diabetes, smoking, alcohol consumption, physical activity, red meat and wheat, BMI, WHR | HR (95%CI) high/low intake (daily vs less than daily): | |
|  |  |  |  |  |  | FV combined  for ♂ & ♀ combined | 0.91 (0.85, 0.97) |
| Du et al. 2017 [16]  The China Kadoorie Biobank Study (CKB)  China | 482 591 men and women  Age 30-79 | 7 | 9504  Linkage with local disease and death registries, health insurance databases | Administered laptop-based questionnaire on diet  Baseline intake in five frequency levels for total fresh fruit intake | Age, sex, region, education, income, alcohol, smoking, physical activity, survey season, BMI, family history of diabetes, dairy products, meat, preserved vegetables | HR (95% CI) highest/lowest intake (daily vs never/rarely): | |
|  |  |  |  |  |  | Total fruit  for ♂ & ♀ combined | 0.88 (0.83–0.93) |
| Auerbach et al. 2017 [17]  The Women’s Health Initiative (WHI)  USA | 114 219 postmenopausal women  Age 50-79 | 7.8 | 11488  Self-reported, validated by medication inventory and fasting plasma glucose levels | 122 semi-quantitative FFQ  Baseline intake in servings/day for total whole fruit intake and fruit juice intake | Age, education level, race/ethnicity, smoking status, physical activity, body mass index, hormone replacement therapy status, study arm, and total energy intake | HR (95% CI) highest/lowest quintile: | |
|  |  |  |  |  |  | Total fruit | 1.00 (0.94–1.06) |
| Alperet et al. 2017 [18]  Singapore Chinese Health Study (SCHS)  Singapore | 45 411 men and women  Age 45-74 | 10.9 | 5207  Self-reported, validated by linkage with a nationwide hospital-based discharge database and supplementary questionnaire | 165 semi-quantitative FFQ  Baseline intake in servings per week or per day for total fruit, fruit subtypes and fruit juice intake | Age, sex, dialect group, year of baseline interview, total daily energy intake, physical activity, education, smoking, alcohol intake, BMI, total vegetable intake, unsweetened soy intake, saturated fat intake, dairy intake, soft drink intake, coffee intake, black and green tea intake, fruit- and vegetable juice intake, mutually adjusted for individual fruits | HR (95% CI) highest/lowest intake (≥3 servings/day vs <1 serving/week): | |
|  |  |  |  |  |  | Total fruit ♂  Total fruit ♀ | 1.33 (1.04, 1.71)  0.88 (0.71, 1.11) |
| Mamluk et al. 2017 [19]  EPIC Elderly study and NIH-AARP  Spain, Greece, the Netherlands, Sweden and USA | EPIC Elderly: 20,629 men and women  NIH-AARP: 401,909 men and women  Age ≥50 | EPIC Elderly: 11.8  NIH-AARP: 10.6 | EPIC Elderly: 1567  NIH-AARP: 22782  Self-reported through questionnaires or interviews | EPIC Elderly: 200 item FFQ, 24h recalls and in some centres 7 or 14 days records of intake  NIH-AARP: 124 item FFQ  Baseline intake in portions/day for fruit and vegetables and in portions/ week for GLV and cabbage | Age, sex, BMI, habitual vigorous physical activity, energy intake, alcohol consumption, education, smoking | OR (95%CI) highest/lowest intake (≥4 vs <1.5 portions/d): | |
|  |  |  |  |  |  | Total fruit (EPIC Elderly)  Total fruit (NIH-AARP)  Total vegetables (EPIC Elderly)  Total vegetables (NIH-AARP)  for ♂ & ♀ combined | 1.01 (0.80-1.28)  0.95 (0.91-0.99)  1.05 (0.79-1.37) _  0.92 (0.87-0.97) |
| Mursu et al. 2014 [20]  Kuopio Ischaemic Heart Disease Risk Factor Study  Finland | 2,332 men  Age 42-60 | 19.3 | 432  Self-administered questions for a physician-set diagnosis, fasting blood glucose measurement 2-h OGTT and record linkage to hospital and social insurance registries | 4-day food records  Baseline intake in g/day for total fruit, total vegetables, FV combined, fruit and vegetable subgroups | Age, BMI, WHR, physical activity, smoking, total energy intake, examination years, education, alcohol, family history of diabetes | HR (95% CI) highest/lowest quartile: | |
|  |  |  |  |  |  | FV combined  Total fruit  Total vegetables | 0.76 (0.57, 1.02)  0.98 (0.75, 1.29)  0.81 (0.61, 1.07) |
| Qiao et al. 2014 [21]  Women’s Health Initiative (WHI)  USA | 154,493 women  Age 50-79 | 7.6 | 10307  Self-reported, validated by medication and laboratory data | 122 item FFQ  Baseline intake in servings/day for total fruit, total vegetable intake | Age, education, cigarette smoking, BMI, waist/hip ratio, physical activity, log (daily energy intake), family history of diabetes, study arms and hormone therapy use | HR (95% CI) high/low intake (≥3.01 vs <3.01 servings/d for V; ≥2.57 vs < 2.57 servings/d for F): | |
|  |  |  |  |  |  | Total vegetables  Total fruit | 1.10 (0.96, 1.26)  0.99 (0.93, 1.06) |
| Kurotani et al. 2013 [22]  Japan Public Health Center-based Prospective Study  Japan | 48,437 men and women  Age 45-75 | 5 | 896  Self-reported | 147 item FFQ  Baseline intake in g/day for total fruit, total vegetables, FV combined, and specific vegetable or fruit items | Age, BMI, physical activity, smoking, area, alcohol, history of hypertension, total energy intake, family history of diabetes, coffee consumption, Mg and Ca intake | OR (95% CI) highest/lowest quartile: | |
|  |  |  |  |  |  | FV combined ♂  FV combined ♀  Total fruit ♂  Total fruit ♀  Total vegetables ♂  Total vegetables ♀ | 0.93 (0.67, 1.29)  1.04 (0.69, 1.55)  0.94 (0.71, 1.26)  1.04 (0.73, 1.48)  0.81 (0.59, 1.13)  0.99 (0.66, 1.47) |
| Cooper et al. 2012 [23]  EPIC-InterAct study  France, Germany, Netherlands, UK, Italy, Sweden, Denmark, Spain | 16,154 men and women  Age 40-79 | 11 | 10821  Self-reported  Linkage to primary & secondary care registers, medication use, mortality data | Country specific dietary questionnaires  Baseline intake in g/day for total fruit, total vegetables, FV combined, fruit and vegetable sub-types | Age (as underlying time scale), sex, education level, BMI, physical activity, smoking, total energy intake, alcohol, study centre; for analysis with fruit also adjusted for vegetables and vice versa | HR (95% CI) highest/lowest quartile: | |
|  |  |  |  |  |  | FV combined  Total fruit  Total vegetables  for ♂ & ♀ combined | 0.90 (0.80, 1.01)  0.89 (0.76, 1.04)  0.94 (0.84, 1.05) |
| Montonen et al. 2005 [24]  Finnish Mobile Clinic Health Examination Survey  Finland | 4,304 men and women  Age 40-69 | 23 | 383  Identification from the Social Insurance Institution’s nationwide register of persons receiving drug reimbursement | Dietary history interview  Baseline intake in g/day for total fruit and berries, total vegetables and green vegetables. | Age, sex, BMI, smoking, total energy intake, family history of diabetes, geographical area | RR (95% CI) highest/lowest quartile: | |
|  |  |  |  |  |  | Total fruit & berries  Total vegetables  for ♂ & ♀ combined | 0.69 (0.51, 0.92)  0.77 (0.57, 1.03) |
| Liu et al. 2004 [25]  Women’s Health Study  USA | 38018 women  Age ≥ 45 | 8.8 | 1614  Self-reported Confirmed by ADA criteria | 131-item FFQ  Baseline intake in servings/day for total fruit, total vegetables, FV combined and separate for GLV, cruciferous, dark yellow, other | Age, BMI, physical activity, smoking, total energy intake, alcohol, family history of diabetes, cholesterol, hypertension | RR (95% CI) highest/lowest quintile: | |
|  |  |  |  |  |  | FV combined  Total fruit  Total vegetables | 1.04 (0.87, 1.25)  0.97 (0.82, 1.16)  1.03 (0.86, 1.23) |
| Hodge et al 2004 [26]  Melbourne Collaborative Cohort Study (MCCS)  Australia | 31,641 men and women  Age 27-75 | 4 | 365  Self-reported/ confirmation by doctor | 121 item FFQ  Baseline intake in times/day for total fruit and total vegetables | Age, sex, country of birth, physical activity, family history of diabetes, alcohol, education, weight change in the last 5 years, energy intake, BMI, WHR | OR (95%CI) highest/lowest quantile: | |
|  |  |  |  |  |  | Total fruit  Total vegetables  for ♂ & ♀ combined | 0.85 (0.59, 1.22)  0.88 (0.60, 1.28) |
| Ford et al. 2001 [27]  NHANES  USA | 9,665 men and women  Age 25-74 | 20 | 1018  Self-reported, hospitalization records, death certificate | Single 24-hour recall  Baseline intake in servings/day for FV combined | Age, sex, BMI, physical activity, smoking, alcohol, cholesterol, antihypertensive medication, systolic blood pressure, education | HR (95% CI) highest/lowest tertile: | |
|  |  |  |  |  |  | FV combined ♂  FV combined ♀ | 1.14 (0.67, 1.93)  0.61 (0.42, 0.88) |
| Meyer et al. 2000 [28]  Iowa Women’s Health Study  USA | 35,988 women  Age 55-69 | 6 | 1141  Self-reported | 127 item FFQ  Baseline intake in servings/week for total fruit, total vegetables and FV combined | Age, BMI, WHR, physical activity, smoking, total energy intake, alcohol, education | RR (95% CI) highest/lowest quintile: | |
|  |  |  |  |  |  | FV combined  Total fruit  Total vegetables | 1.05 (0.84, 1.31)  1.14 (0.93, 1.39)  1.07 (0.86, 1.32) |
| Colditz et al. 1992 [29]  Nurses Health Study (1980)  USA | 84,360 women (nurses)  Age 34–59 | 6 | 702  Self-reported follow-up questionnaire | 61-item FFQ  Baseline intake in servings/day for total fruit and total vegetables | Age, BMI, weight change, alcohol, energy intake | HR (95% CI) highest/lowest quintile: | |
|  |  |  |  |  |  | Total vegetables  Total fruit | 0.76 (0.50–1.16)  1.15 (0.72-1.84) |

ADA, American Diabetes Association; FFQ, food frequency questionnaire; FV, fruit and vegetables; GLV, green leafy vegetables; ♂: men, ♀: women. ^a^ the associations are presented for the highest compared to the lowest intake level. For a review including FV subtypes and juices, please see the meta-analysis of Halvorsen et al. [30].

**Supplementary Fig. 1.** **Design of the Stockholm Diabetes Prevention Program from baseline to Follow-up 2**

Postal questionnaire to all men and women aged 35-56 years, residing within 5 municipalities in Stockholm

Men: 12,952 Women: 19,416

Responders:

**Men: 10,236 (79%)**

**Women: 16,481 (85%)**

Excluded ^a^

Men: 4,801 (47%)

Women: 8,178 (50%)

No FHD

**Men: 3,329**

**Women: 4,296**

FHD

**Men: 2,106**

**Women: 3,583**

Gestational diabetes

**Women: 424**

Excluded ^b^

Women: 466

Excluded ^c^

Men: 34

Women: 126

*Health examination 2*

**Baseline study group 7,948**

**Men: 3,128 Women: 4,820**

FHD: 52% FHD: 54%

**Follow-up 1 study group: 5,719 (73%)**

**Men: 2,384 Women: 3,335**

FHD: 57% FHD: 58%

***Follow-up period***

*Health examination 1*

Baseline study

Men 1992-94

Women 1996-98

Excluded ^d^

Men: 382

Women: 456

Invitation letter to baseline study group

No wish to participate or unreachable

Men: 362

Women: 1029

SDPP Follow-up 1

Men 2002-04

Women 2004-06

***Follow-up period***

Excluded ^e^

Men: 245

Women: 235

Invitation to baseline study group

**Follow-up 2 study group: 3627 (46%)**

**Men: 1474 Women: 2153**

FHD: 58% FHD: 60%

*Health examination 2*

No wish to participate or unreachable

Men: 1409

Women: 2432

SDPP Follow-up 2

Men and women

2014-2017

FHD, family history of diabetes; NGT, normal glucose tolerance; T2D, type 2 diabetes.

^a^ Excluded due to already known diabetes, foreign origin, unclear or insufficient FHD.

^b^ Excluded due to financial reasons (women born 1952-1961 and in the last third of each month)

^c^ Excluded due to uncertain heredity, incomplete examination, pregnancy, breast feeding or medical reasons and 1 subject due to withdrawal of informed consent

^d^ Excluded from invitation to 1^st^ follow-up due to T2D diagnosis at baseline, moved outside of Stockholm county or deceased.

^e^ All men and women included at baseline study group were invited to participate in the 2^nd^ follow-up, independently of whether they participated in the 1^st^ follow-up, excluding those that died (n=480).

| **Supplementary Table 2. Hazard ratios (95% CI) estimated by cox regression analysis for the association between baseline fruit and vegetable intake and risk of diabetes/prediabetes at follow-up (N= 6961 and 5997 in diabetes and prediabetes analyses respectively)** | | | | | | | | | |
| --- | --- | --- | --- | --- | --- | --- | --- | --- | --- |
|  | **T2D n=1024 cases (from NGT/prediabetes)** | | | | | | | |  |
|  | **Women** | | | |  | **Men** | | | *P-interaction* **^a^** |
| **Tertiles** | 1 | | 2 | 3 |  | 1 | 2 | 3 |  |
| **Total FVI** (median) | 178 | | 329 | 536 |  | 106 | 215 | 372 |  |
| Cases  Person-years | 160  28115 | | 153  28072 | 180  28062 |  | 198  18714 | 185  19746 | 148  19447 |  |
| Model 1 | 1.00 | | 0.92 (0.74, 1.15) | 1.04 (0.84, 1.29) |  | 1.00 | 0.87 (0.71, 1.07) | 0.67 (0.54, 0.83) | 0.007 |
| Model 2 | 1.00 | | 1.03 (0.82, 1.29) | 1.23 (0.98, 1.55) |  | 1.00 | 0.98 (0.79, 1.20) | 0.77 (0.62, 0.97) | 0.005 |
| Model 3 | 1.00 | | 0.99 (0.79, 1.25) | 1.11 (0.88, 1.41) |  | 1.00 | 1.01 (0.82, 1.24) | 0.77 (0.62, 0.97) | 0.015 |
| **Total fruit** (median) | 73 | | 180 | 342 |  | 34 | 105 | 223 |  |
| Cases  Person-years | 160  28139 | | 169  28036 | 164  28073 |  | 206  19384 | 167  19120 | 158  19402 |  |
| Model 1 | 1.00 | | 1.02 (0.82, 1.26) | 0.97 (0.78, 1.20) |  | 1.00 | 0.80 (0.65, 0.98) | 0.72 (0.58, 0.88) | 0.086 |
| Model 2 | 1.00 | | 1.10 (0.87, 1.37) | 1.07 (0.84, 1.36) |  | 1.00 | 0.93 (0.75, 1.15) | 0.85 (0.68, 1.06) | 0.108 |
| Model 3 | 1.00 | | 1.07 (0.85, 1.34) | 1.00 (0.79, 1.27) |  | 1.00 | 0.94 (0.76, 1.16) | 0.82 (0.65, 1.03) | 0.206 |
| **Total vegetables** (median) | 69 | | 132 | 230 |  | 44 | 98 | 177 |  |
| Cases  Person-years | 161  27780 | | 155  28636 | 177  27834 |  | 214  18562 | 156  19840 | 161  19506 |  |
| Model 1 | 1.00 | | 0.89 (0.72, 1.12) | 1.02 (0.82, 1.26) |  | 1.00 | 0.69 (0.56, 0.85) | 0.69 (0.56, 0.85) | 0.022 |
| Model 2 | 1.00 | | 1.00 (0.79, 1.26) | 1.18 (0.94, 1.49) |  | 1.00 | 0.77 (0.62, 0.96) | 0.79 (0.64, 0.99) | 0.018 |
| Model 3 | 1.00 | | 1.02 (0.81, 1.29) | 1.14 (0.90, 1.45) |  | 1.00 | 0.81 (0.66, 1.01) | 0.83 (0.66, 1.03) | 0.056 |
|  | **Prediabetes n=862 cases (from NGT)** | | | | | | | |  |
|  | **Women** | | | | | **Men** | | | *P-interaction* **^a^** |
| **Tertiles** | 1 | | 2 | 3 |  | 1 | 2 | 3 |  |
| **Total FVI** (median) | 177 | | 329 | 537 |  | 109 | 216 | 372 |  |
| Cases  Person-years | 128  25130 | | 138  25343 | 154  24761 |  | 153  14952 | 155  16061 | 134  16390 |  |
| Model 1 | 1.00 | | 1.02 (0.81, 1.30) | 1.13 (0.89, 1.43) |  | 1.00 | 0.92 (0.73, 1.15) | 0.74 (0.58, 0.93) | 0.024 |
| Model 2 | 1.00 | | 1.04 (0.81, 1.34) | 1.23 (0.96, 1.58) |  | 1.00 | 0.96 (0.76, 1.21) | 0.81 (0.64, 1.04) | 0.010 |
| Model 3 | 1.00 | | 1.00 (0.78, 1.28) | 1.15 (0.90, 1.48) |  | 1.00 | 0.99 (0.78, 1.24) | 0.80 (0.63, 1.03) | 0.012 |
| **Total fruit** (median) | 73 | | 180 | 340 |  | 34 | 105 | 225 |  |
| Cases  Person-years | 131  25193 | | 139  25032 | 150  25009 |  | 163  15390 | 140  15789 | 139  16224 |  |
| Model 1 | 1.00 | | 1.02 (0.80, 1.29) | 1.08 (0.86, 1.37) |  | 1.00 | 0.80 (0.64, 1.00) | 0.74 (0.59, 0.93) | 0.059 |
| Model 2 | 1.00 | | 1.00 (0.78, 1.29) | 1.11 (0.86, 1.43) |  | 1.00 | 0.90 (0.71, 1.13) | 0.85 (0.66, 1.08) | 0.048 |
| Model 3 | 1.00 | | 0.98 (0.76, 1.25) | 1.05 (0.82, 1.36) |  | 1.00 | 0.88 (0.70, 1.11) | 0.81 (0.63, 1.03) | 0.058 |
| **Total vegetables** (median) | 69 | 132 | | 229 |  | 44 | 99 | 176 |  |
| Cases  Person-years | 120  24847 | | 147  25674 | 153  24712 |  | 165  14673 | 132  16629 | 145  16100 |  |
| Model 1 | 1.00 | | 1.15 (0.90, 1.47) | 1.20 (0.95, 1.53) |  | 1.00 | 0.70 (0.56, 0.88) | 0.76 (0.61, 0.95) | 0.004 |
| Model 2 | 1.00 | | 1.17 (0.91, 1.51) | 1.27 (0.98, 1.64) |  | 1.00 | 0.74 (0.59, 0.94) | 0.84 (0.66, 1.07) | 0.002 |
| Model 3 | 1.00 | | 1.19 (0.93, 1.53) | 1.25 (0.96, 1.61) |  | 1.00 | 0.76 (0.60, 0.96) | 0.86 (0.68, 1.09) | 0.003 |

Model 1: age, FHD; Model 2: Model 1 + education, SEI, high blood pressure, physical activity, smoking, alcohol, wholegrain intake,

yoghurt/sour milk intake, total fruit intake for total vegetable intake analyses and vice versa; Model 3: Model 2 + BMI

| **Supplementary Table 3. Rates of loss to follow-up from baseline in prediabetes analyses, displayed by tertiles of baseline FVI, total fruit and total vegetable intake** | | | | | | | |
| --- | --- | --- | --- | --- | --- | --- | --- |
|  |  | **Baseline sample prediabetes** | | **Lost at follow-up 1**^a^ | | **Lost at follow-up 2**^a^ | |
| Baseline tertiles |  | Women | Men | Women | Men | Women | Men |
| Total FV | 1 | 1278 | 722 | 431 (34%) | 136 (19%) | 657(51%) | 326 (45%) |
|  | 2 | 1278 | 721 | 382 (30%) | 142 (20%) | 623 (49%) | 316 (44%) |
|  | 3 | 1277 | 721 | 323 (25%)^*^ | 151 (21%) | 579 (45%)^*^ | 297 (41%) |
| Total fruit | 1 | 1278 | 742 | 435 (34%) | 150 (20%) | 664 (52%) | 333 (45%) |
|  | 2 | 1284 | 717 | 372 (29%) | 131 (18%) | 615 (48%) | 311 (43%) |
|  | 3 | 1271 | 705 | 329 (26%)^*^ | 148 (21%) | 580 (46%)^*^ | 295 (42%) |
| Total vegetables | 1 | 1285 | 722 | 429 (33%) | 144 (20%) | 652 (51%) | 324 (45%) |
|  | 2 | 1271 | 724 | 375 (30%) | 148 (20%) | 612 (48%) | 314 (43%) |
|  | 3 | 1277 | 718 | 332 (26%)^*^ | 137 (20%) | 595 (47%) | 301 (42%) |

^a^ Values are numbers (rates) of loss to follow-up from baseline in each tertile. ^*^Significant different rates of loss to follow-up from

baseline among tertiles within the same FV category. Analyses performed with chi-square tests, significance level p<0.05.
